# The 100 most-cited articles on cardiovascular diseases from Mainland China

**DOI:** 10.1186/s12872-015-0083-4

**Published:** 2015-08-28

**Authors:** Yuan-hui Liu, Sheng-qi Wang, Jin-hua Xue, Yong Liu, Ji-yan Chen, Guo-feng Li, Ning Tan

**Affiliations:** Department of Cardiology, Guangdong Cardiovascular Institute, Guangdong General Hospital, Guangdong Academy of Medical Sciences, Guangzhou, 510100 China; Department of Pharmacy, Nanfang Hospital, Southern Medical University, Guangzhou, 510515 China; Department of Pathophysiology, School of Basic Medical Sciences, Southern Medical University, Guangzhou, 510515 China; Department of Physiology, School of Basic Medical Sciences, Gannan Medical University, Ganzhou, 341000 China

## Abstract

**Background:**

China, as a rapidly developing country with the largest population of cardiologist in the world, has an increasing importance in the field of cardiology. However, the quantity and quality of research production in the field of cardiology is unclear.

**Aims:**

To analyze the characteristics of the high-level articles published on cardiovascular diseases in Mainland China, and to provide information about achievements and development in cardiovascular research.

**Methods:**

We searched the Science Citation Index Expanded for citations of cardiovascular articles originating in mainland China from 2004 to 2015. For the 100 most frequently cited articles (T100), we evaluated the number of citations, publication time, province of origin, journal, impact factor, topic or subspecialty of the research, and publication type.

**Results:**

The most frequently cited article received 703 citations at the most, while 50 at the least (mean 91.6 citations per article). T100 originated from 16 provinces, the plurality (*n* = 34) being from the Beijing. Sixty-seven percent were published during 2006–2009. The publications were in 29 different journals of which Circulation published the most (*n* = 14). Leading general medical journals Journal of the American Medical Association (*n* = 1), Lancet (*n* = 0) and New England Journal of Medicine (*n* = 0) featured only 1 published article, despite their extremely high impact factors. Of the T100 articles, there were 50 basic researches, 44 clinical researches, 5 meta-analyses and 1 review article. Clinical researches had the highest mean citations (mean 102.6 citations per article).

**Conclusions:**

This study provides a historical perspective on the scientific progress, and the trends in cardiovascular medicine in Mainland China.

**Electronic supplementary material:**

The online version of this article (doi:10.1186/s12872-015-0083-4) contains supplementary material, which is available to authorized users.

## Background

Citation analysis is one of the most widely employed methods of bibliometrics. It is used to determine the impact factor (IF) of a journal and useful for evaluating the impact of an article on the scientific community. Academic institutions, funding agencies, and the public are now increasingly interested in assessing the research quality and productivity of individual researchers by citation analysis [1]. Therefore, there have been numerous attempts to identify the most highly cited articles in various medical specialties including traumatic brain injury [2], radiology [3], hypospadiology [4], microsurgery [5], orthopaedic surgery [6], hypertension [7] and cardiac surgery [8].

Cardiovascular disease has evolved substantially in the last few decades. It is becoming an increasingly common disease in China, which, with more than 1.3 billion citizens, is the world’s most populous developing country. As China has grown economically, it has experienced an epidemiological transition, with mortality due to cardiovascular diseases, such as ischaemic heart disease, more than doubling during the past two decades to more than 1 million deaths per year [9]. Research originating from Mainland China related to cardiovascular diseases has made great advances in recent decades, gaining increasing importance and attention in the world community. However, there is still a lack of knowledge regarding the quality of the scientific yield in this area. Therefore, the present study aimed to analyze the characteristics of the 100 top-cited articles published on cardiovascular diseases in Mainland China, and to provide information about achievements and development in cardiovascular research over the past ten years.

## Methods

In March 2015, we performed a citation search for published articles on cardiovascular diseases from 2004 to 2015, using the Science Citation Index Expanded of the ISI Web of Science (Thomson Reuters, Philadelphia, PA, USA). There were 107 journals in our investigation under the subject category of “cardiology, cardiovascular, and heart.” in Journal Citation Reports for 2014. In addition, we searched three leading clinical medicine journals, the New England Journal of Medicine, the Lancet, and the Journal of the American Medical Association. Finally, a total of 110 journals were used to identify the most-cited articles in our study. There were no restrictions on the study types, but only articles written in the English language were included.

All included journals were collected in a single search of the Web of Science. A filter for “Countries/territories” was applied by selecting “Peoples R China” to include articles originating only from Mainland China. After all indexed published articles had been retrieved, the results were sorted using the option “Times cited”, which provided us with a list of all the articles published in a specific journal ranked by citation count. Each article was then evaluated, and articles having first author and first address in Mainland China were included, while those that originated outside Mainland China, or had one or more Chinese co-authors but not the first author were excluded.

Of the total articles remaining, the top 100 according to the number of citations (T100) were further analyzed by two independent reviewers. Applying the same methods used in previous similar studies [10], the following data were compiled: journal name, title, number of citations, decade published, number of authors, publication year, impact factor, province of origin, and topic or subspecialty of the research. In addition, two independent reviewers reviewed titles and abstracts to determine the study types: meta-analysis, basic research, or clinical research (observational or randomized control trials). Another reviewer resolved any disagreements regarding article eligibility. We also reviewed the association between journal IF and the number of articles, and the numbers of citations included in the top 100 articles. The IFs of the journals were cross-referenced with the 2014 edition of Journal Citation Reports (JCR): Science Edition (1945–2013).

### Statistical analysis

Data were expressed as median and interquartile range. The Wilcoxon rank sum test was performed to evaluate differences between groups. The Spearman test was used to evaluate the strength and direction of the linear relation between the IF of the journal and the numbers of top 100 cited articles or citations included in the list. All data analyses were performed using SAS, version 9.2 (SAS Institute, Cary, NC, USA). All probability values were two-tailed, and the statistical significance was defined as *p* < 0.05.

## Results

**Fig. 1:**
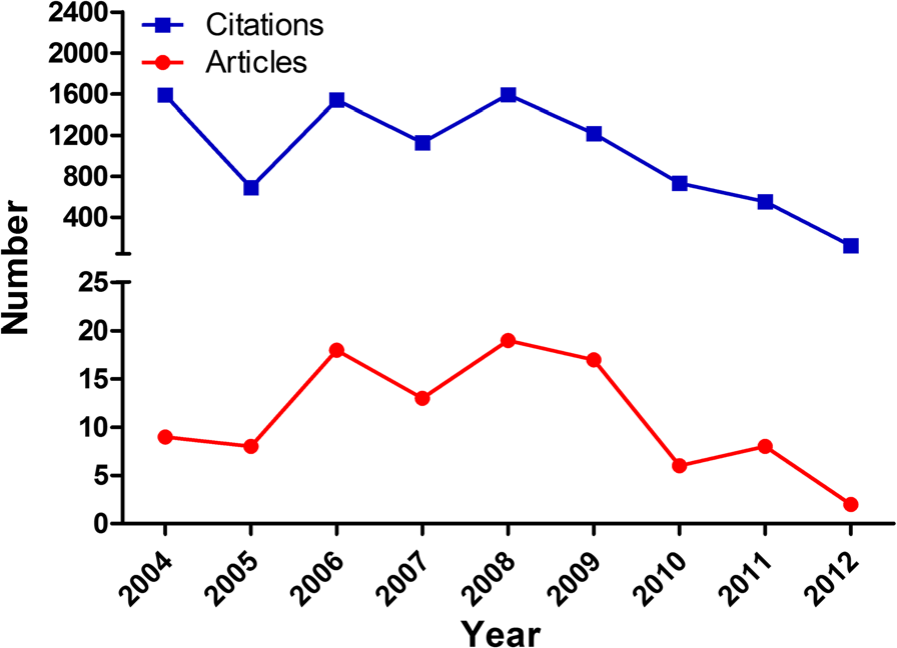
Numbers of articles published in each year and the corresponding numbers of citations in each year

The T100 articles are listed in Table 1 and Additional file 1. (1–20 in Table 1; A complete list (21–100) is given in Additional file 1 as supplementary data on-line). The number of citations for the T100 articles ranged from 50 to 703, while only four papers were cited over 200 times. Of the T100 articles, 67 % were published in the 4-year period from 2006 to 2009 (Fig. 1), while none were published in the latest three years (2013–2015). The mean number of citations per year was 216 (range 161 to 266) for these four years.

**Table 1:** Bibliometric information associated with the Top 20 of the Top 100 most frequently cited articles on cardiovascular diseases (T100) from mainland china

Rank	Author	Title	Journal	Year	Times Cited	PMID
1	Chen, SL et al.	Effect on left ventricular function of intracoronary transplantation of autologous bone marrow mesenchymal stem cell in patients with acute myocardial infarction	American Journal of Cardiology	2004	703	15219514
2	Wang, Guo-Kun et al.	Circulating microRNA: a novel potential biomarker for early diagnosis of acute myocardial infarction in humans	European Heart Journal	2010	346	20159880
3	Liu, J et al.	Predictive value for the Chinese population of the Framingham CHD risk assessment tool compared with the Chinese multi-provincial cohort study	Journal of The American Medical Association	2004	273	15173150
4	Hu, Xin yang et al.	Transplantation of hypoxia-preconditioned mesenchymal stem cells improves infarcted heart function via enhanced survival of implanted cells and angiogenesis	Journal of Thoracic and Cardiovascular Surgery	2008	207	18374759
5	Zhang, Qing-jun et al.	Endothelium-specific overexpression of class III deacetylase SIRT1 decreases atherosclerosis in apolipoprotein E-deficient mice	Cardiovascular Research	2008	165	18689793
6	Wu, Yang feng et al.	Prevalence, Awareness, Treatment, and Control of Hypertension in China Data from the China National Nutrition and Health Survey 2002	Circulation	2008	158	19106390
7	Ma, J et al.	Time course of myocardial stromal cell-derived factor 1 expression and beneficial effects of intravenously administered bone marrow stem cells in rats with experimental myocardial infarction	Basic Research In Cardiology	2005	156	15754085
8	Xu, FP et al.	Leptin induces hypertrophy via endothelin-1-reactive oxygen species pathway in cultured neonatal rat cardiomyocytes	Circulation	2004	154	15313952
9	Li, Xiao Hong et al.	Bone marrow mesenchymal stem cells differentiate into functional cardiac phenotypes by cardiac microenvironment	Journal of Molecular and Cellular Cardiology	2007	131	16919679
10	Wang, TZ et al.	Cell-to-cell contact induces mesenchymal stem cell to differentiate into cardiomyocyte and smooth muscle cell	International Journal of Cardiology	2006	130	16122823
11	Liu, Tong et al.	Association between C-reactive protein and recurrence of atrial fibrillation after successful electrical cardioversion-A meta-analysis	Journal of The American College of Cardiology	2007	129	17433956
12	Wan, Yi et al.	Anticoagulation Control and Prediction of Adverse Events in Patients With Atrial Fibrillation A Systematic Review	Circulation-Cardiovascular Quality and outcomes	2008	127	20031794
13	Ge, J. et al.	Efficacy of emergent transcatheter transplantation of stem cells for treatment of acute myocardial infarction (TCT- STAMI)	Heart	2006	127	16775089
14	Hou, Ying long et al.	Ganglionated plexi modulate extrinsic cardiac autonomic nerve input-Effects on sinus rate, atrioventricular conduction, refractoriness, and inducibility of atrial fibrillation	Journal of The American College of Cardiology	2007	122	17601547
15	Shan, Hong li et al.	Downregulation of miR-133 and miR-590 contributes to nicotine-induced atrial remodelling in canines	Cardiovascular Research	2009	119	19398468
16	Hu, Da-Yi et al.	The relationship between coronary artery disease and abnormal glucose regulation in China: the China Heart Survey	European Heart Journal	2006	115	16984927
17	Min, TQ et al.	Improvement in endothelial progenitor cells from peripheral blood by ramipril therapy in patients with stable coronary artery disease	Cardiovascular Drugs and Therapy	2004	115	15229388
18	Lu, Yan jie et al.	MicroRNA-328 Contributes to Adverse Electrical Remodeling in Atrial Fibrillation	Circulation	2010	112	21098446
19	Wang, LH et al.	TRPV1 gene knockout impairs postischemic recovery in isolated perfused heart in mice	Circulation	2005	111	16314376
20	He, Y et al.	Prevalence of the metabolic syndrome and its relation to cardiovascular disease in an elderly Chinese population	Journal of The American College of Cardiology	2006	110	16630995

Note: See Additional file 1 for the other 80 articles of the T100

**Fig. 2:**
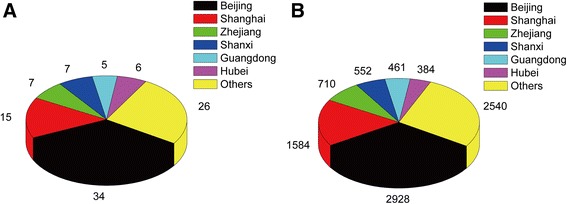
The T100 articles were analyzed in terms of their origin province. **a**: Numbers of articles in each province. **b**: Numbers of citations for the articles in each province

The T100 articles originated from 14 different provinces of Mainland China, a plurality being from Beijing (*n* = 34, 2928 citations), followed by Shanghai (*n* = 15), Zhejiang (*n* = 7), Shanxi (*n* = 7), Hubei (*n* = 6), and Guangdong (*n* = 5), while the remaining provinces had <5 highly cited articles (Fig. 2a-b). Articles originated from Shanghai had the highest mean citations (mean 105.6 citations per article).

The leading first author, with 703 citations, was Chen SL, the next 4 were Wang GK (*n* = 346), Liu J (*n* = 273), Hu XY (*n* = 207), and Zhang QJ (*n* = 165). Among the T100 articles, most of the authors had just 1 highly cited article, and only Chen SL, had 3 highly cited articles as the first author, with a mean of 273 citations.

The T100 articles were published in 29 different journals. The journal with the highest number of T100 articles was Circulation (*n* = 14), followed by Cardiovascular Research (*n* = 10), Journal of the American College of Cardiology (*n* = 10), Atherosclerosis (*n* = 7), Circulation Research (*n* = 7), European Heart Journal (*n* = 7), and International Journal of Cardiology (*n* = 6). However, the general medical journals, Journal of the American Medical Association (*n* = 1), Lancet (*n* = 0) and New England Journal of Medicine (*n* = 0) featured only 1 article in the list, despite their extremely high IFs. The IFs for the journals that published the T100 articles ranged from 1.1 to 30.4. Many of the T100 articles were published in high- IF journals (Table 2), while the journal IF was significantly correlated with the number of T100 articles (r = 0.51, *p* = 0.004), and the number of citations (r = 0.58, *p* = 0.001).

**Table 2:** Journals in which the T100 articles were published

Journal	No. of Articles (Citations)	Impact Factor
Circulation	14(1308)	14.9
Cardiovascular Research	10(787)	5.81
Journal of the American College of Cardiology	10(743)	15.34
Atherosclerosis	7(631)	3.97
Circulation Research	7(483)	11.09
European Heart Journal	7(795)	14.72
International Journal of Cardiology	6(454)	6.18
journal of molecular and cellular cardiology	5(377)	5.22
Journal of Cardiovascular Electrophysiology	4(287)	2.88
American Heart Journal	3(210)	4.56
European Journal of Cardio-Thoracic Surgery	3(199)	2.81
American Journal of Cardiology	2(789)	3.43
American Journal of Physiology-Heart and Circulatory Physiology	1(71)	4.01
Basic Research in Cardiology	2(239)	5.96
Heart	2(204)	6.02
JACC- Cardiovascular Interventions	2(111)	7.44
Journal of Cardiovascular Pharmacology	2(156)	2.11
Journal of Thoracic and Cardiovascular Surgery	2(292)	3.99
Annals of Thoracic Surgery	1(51)	3.63
Cardiology	1(85)	2.04
Cardiovascular Drugs and Therapy	1(115)	2.95
Circulation- Arrhythmia and Electrophysiology	1(61)	5.42
Circulation- Cardiovascular Quality and Outcomes	1(127)	5.04
Clinical Cardiology	1(53)	2.23
Heart Rhythm	1(63)	4.92
International Heart Journal	1(102)	1.13
JAMA- journal of the American Medical Association	1(273)	3.39
Journal of Electrocardiology	1(63)	1.36
Pediatric Cardiology	1(57)	1.55

**Fig. 3:**
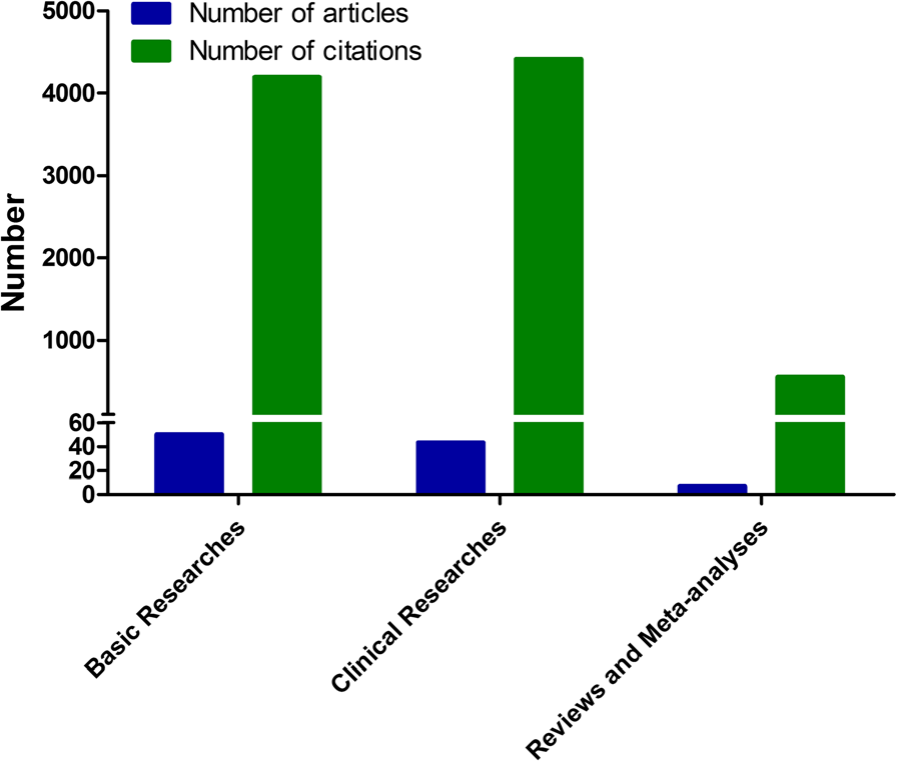
Publication type distributions of the T100 articles and the corresponding numbers of citations

**Fig. 4:**
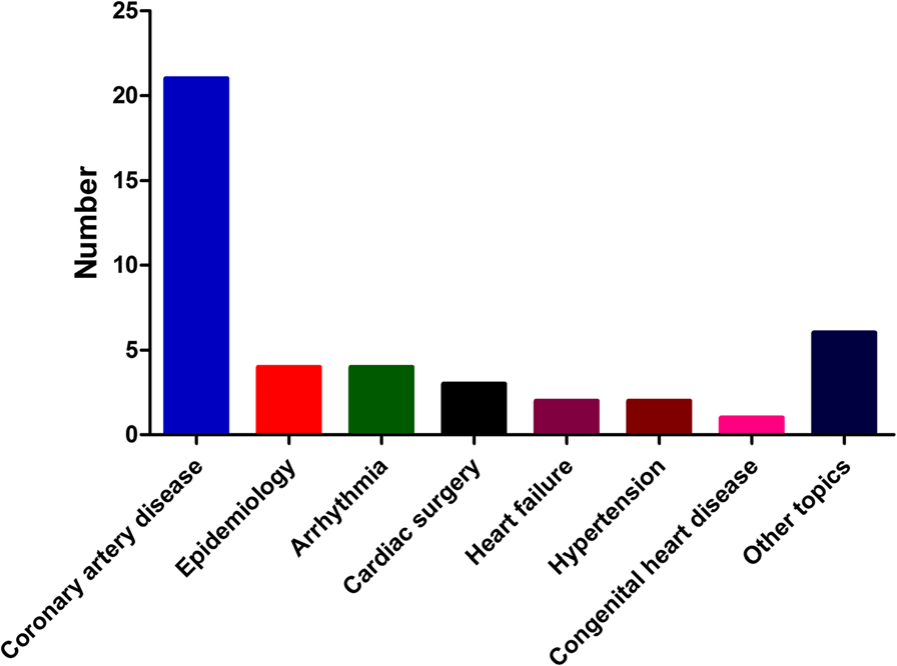
Research topic distributions of the clinical articles

**Fig. 5:**
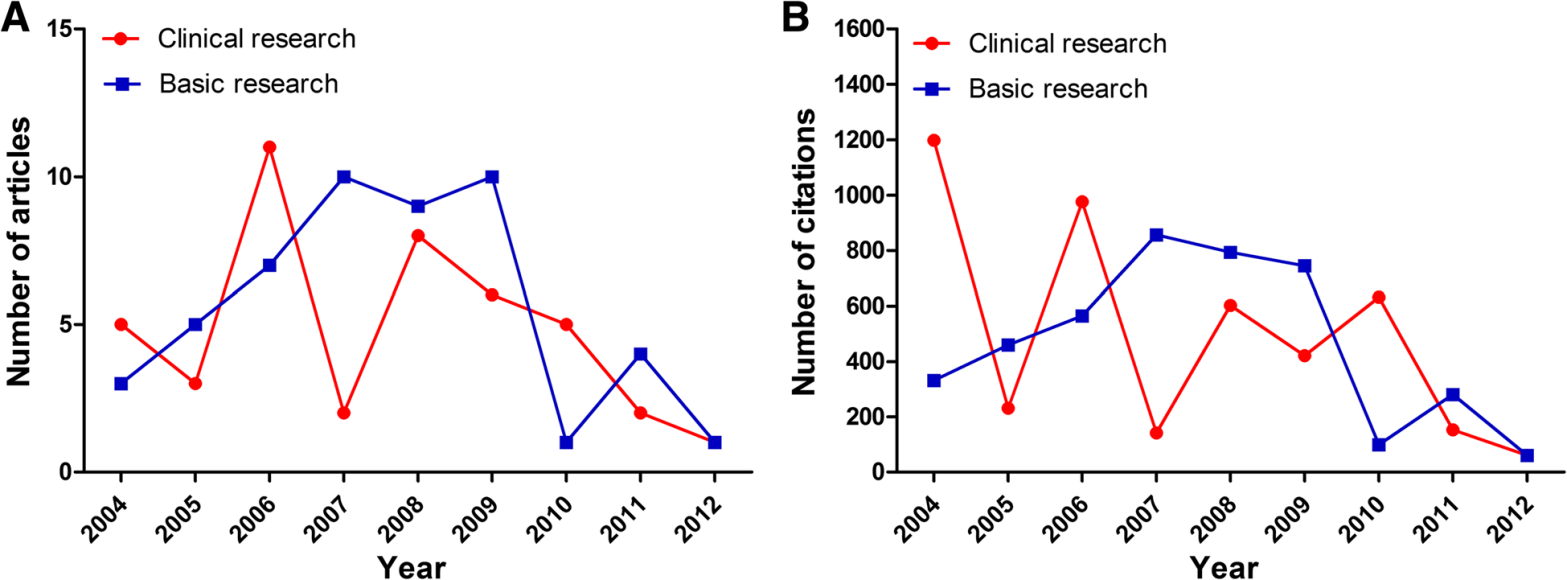
Basic researches and clinical researches were analyzed in terms of their publication time. **a**: Numbers of the indicated articles in each year. **b**: Numbers of citations for the indicated articles in each year

The analysis of the design of the T100 articles found that they included 6 meta-analyses and 1 review, whereas the majority of the article types were basic science studies (50 articles), followed by clinical trials (43 articles) (Fig. 3). Among the 43 clinical articles, 13 were randomized clinical trials and 30 were observational trials. As shown in Fig. 4, these clinical trials were mainly focused on coronary artery disease (21 studies, including 6 on acute coronary syndromes), epidemiology (4 studies), arrhythmia (4 studies), cardiac surgery (3 studies), heart failure (2 studies), hypertension (2 studies), congenital heart disease (1 study), or other topics (6 studies). In terms of time, 5 earliest clinical researches which were published in 2004 were cited the most (239 times per article), while 3 earliest basic researches in 2004 were cited the most (110 times per article) (Fig. 5).

The number of total citations per article ranged from 50 to 703 (mean, 102.6) for clinical articles, and 52 to 207 (mean, 83.8) for basic science. Among the articles with >100 citations, the most common topics were acute myocardial infraction (*n* = 3, 27.3 %) for clinical articles and stem cells (*n* = 4, 33.3 %) for basic science.

## Discussions

The present study is the first to identify, rank and characterize the T100 cited articles from the past ten years in Mainland China in the field of cardiovascular disease. The results offer invaluable insight into the evolution of popular opinion in the field of cardiovascular disease, which has undergone considerable change over the years around the world.

Citation analysis can reflect the articles’ influence in a specific field, help authors recognize important advances in some research area, and add useful perspective on historical developments in our specialty. In addition, journals often use it to attract manuscripts with high citation potential. Furthermore, an understanding of the characteristics inherent to highly cited work would be helpful for young researchers who wish to publish effectively. To date, there are increasing numbers of articles credited as “the most cited” or “top cited” in various medical fields. According to a report on Chinese Cardiovascular Disease in 2013, there were approximately 2.9 billion patients with cardiovascular diseases, and the associated mortality increased from 240.03/10 million in 2004 to 268.9/10 million in 2010 [11]. However, there is little information in the literature concerning the citation of articles about cardiovascular diseases in China. Recently, Shuaib et al. conducted a study to provide insight into the citation frequency of top cited articles published in cardiovascular medicine [12]. Unfortunately, however, no cardiovascular articles from China were included in that analysis. Therefore, it is necessary to analyze the top article citations in cardiovascular disease from Mainland China in the past ten years, which has seen great advances and changes in cardiovascular diseases over that period, to help readers or authors recognize the quality of the research work, discoveries, and the trends steering cardiology.

Fourteen cities contributed to the T100 cited articles, led by Beijing, the capital of China, followed by Shanghai. This confirms the important influence of both cities in relation to cardiovascular diseases and research in Mainland China. This was in concordance with many other citation analyses of other medical specialties from Mainland China, which reported that most studies that made the T100 list came from Beijing or Shanghai. The reason for this may be that these two cities have large numbers of cardiovascular communities and sufficient financial support from the country to perform basic or clinical research.

The IF represents the citation frequency of the average article published in a given journal in a particular year, and is always used to determine the quality of scientific or biomedical journals. Authors would prefer to send their research papers to journals with a high IF. In turn, this gives those journals a greater chance to receive high quality research articles, which will be cited more frequently after being published [13–15]. In accordance with the research performed by Shuaib et al. [12], we also demonstrated that the IF was positively correlated with the number of T100 articles, and the number of citations. Although the evaluation of journals based on their IF involves potential bias and is under debate, there is presently no perfect or even better method for evaluating the merit of a scientific paper, and the IF and citation total are generally accepted as the best criteria.

Our results demonstrated that 38 % of these T100 cited articles were published in Circulation, the Journal of the American College of Cardiology, Circulation Research, or the European Heart Journal, all of which had an IF >11. However, the so-called high IF general medical journals, such as the Journal of the American Medical Association, The Lancet and The New England Journal of Medicine, only contributed 1 article to the list of T100 articles. In addition, journals with relatively lower IF also published highly cited articles (e.g. the 1st and 24th T100 journals, with IF of 3.4 and 5.8 respectively). This indicates a growing trend towards publishing highly influential articles in specialty journals that are dedicated solely to research into cardiovascular diseases, rather than in general medical journals. The present result was in accordance with other bibliometric analyses demonstrating that highly influential reports are often published in specialty journals [16–18].

Generally, with increasing age, each article has more time to be cited, but in this analysis, most of the T100 articles (67 %) were published between 2006 and 2009. Among the articles with >100 citations, 62 % were published during this period. The present result was in agreement with the recent research by Shuaib et al. [12]. Those investigators indicated that 42 % of the T100 cited articles, a lower percentage than for articles from China in the same period, were published between 2006 and 2010 around the world; however, their results excluded research articles from China.

Among the clinical articles, it was observed that a large majority of the most highly cited articles in cardiovascular medicine were focused on coronary artery disease. This was in accordance with the morbidity of CAD according to the report on Chinese Cardiovascular Disease in 2013 [11], which demonstrated that in the past 10 years, the mortality of CAD continued to increase, up to 96/10 million in urban and 76/10 billion in rural areas. The high morbidity and mortality of CAD make it easy to recruit participants in research studies. However, although the prevalence of congenital heart diseases is increasing dramatically (about 200 millions at present), there was only 1 related study in the list. In addition, cardiac tumors, and cardiomyopathies were virtually unpresented in the list. Therefore, the present list could help point out the topics and subspecialties that have not been given due consideration, and should be the focus of future research.

China’s heavy burden of disease can actually be viewed as a fruitful clinical resource for physicians to design clinical research and collect clinical data. However, most T100 cited papers were focused on basic research. Furthermore, no guidelines were reported in the present analysis for Mainland China. This differed from other analyses, in which the leading type of T100 research report was clinical studies, rather than basic studies [7, 19]. The status of clinical research in Mainland China might be attributed to several reasons. First, many physicians do not have the skills, such as a familiarity with statistical analysis, to understand and carry out clinical research to an international standard. In addition, many hospitals in Mainland China do not have mature, integrated platforms or systems for clinicians to perform clinical research. In addition, insufficient funding, and a lack of available time are other important barriers to performing the clinical researches. Furthermore, many clinicians believe that clinical research conflicts with the accumulation of experience in clinical practice [20]. However, the environment is changing with respect to these issues. On June 8, 2013, the Chinese Government officially launched 13 national clinical research centers, which are led by prominent hospitals with strong clinical experience and research capacities, including two for cardiovascular diseases [21]. These centers aim to lead hospitals across China to strengthen their clinical research capacity, and to organize multicenter clinical research projects. This development is an important step in building China’s clinical research capacity. In the future, there will be a growing amount of important clinical research done by Chinese physicians, and these will not only contribute to Chinese clinical practice and health policies, but will also add to the knowledge that is essential for international guidelines. Recently, more clinical research articles originating from the mainland China, such as those by Huo Y et al. [22], Li J et al. [23] and Han Y et al. [24], have been published in high IF journals and provide important data from Chinese and international institutes.

### Limitations

The present study had several limitations. First, despite a meticulous search in the web of science database, there is a chance that some studies were missed. Furthermore, we only analyzed articles published in the past 10 years, so it is likely that some truly “classic” articles were excluded. It is also true that, analysis using other database platforms such as Scopus or the PubMed Central database, might lead to a different list. Second, this type of study usually favors older published articles, while recently published high quality studies would not be included, a limitation which is related to the effect of time on citations. Third, the value of contributions to the field can’t be quantified only by the number of citations. Therefore, papers that are important and influential, but have a lower citation frequency, might have been missed. Last, the language of publication plays a major role, with a bias towards articles published in English language journals.

## Conclusions

Our analysis provides a summary of the most influential studies on cardiovascular disease originating from Mainland China from 2004 to 2015, and highlights areas of research that require further investigation and development. In addition, Our analysis also provide an insight into the citation frequency of the top cited articles published in cardiovascular medicine to shed light on the quality of the work, discoveries, and the trends steering cardiology in Mainland China.

## Additional file

Additional file 1:
**Bibliometric information associated with the other 80 articles of the T100.** (DOCX 44 kb)
