# Supplementary material for: The 100 most-cited articles on cardiovascular diseases from Mainland China
Source: BMC Cardiovasc Disord. 2015 Aug 28;15:94. doi: 10.1186/s12872-015-0083-4 (PMC4551365; doi:10.1186/s12872-015-0083-4)
Supplement: Additional file 1: — Bibliometric information associated with the other 80 articles of the T100. (DOCX 44 kb) [file 12872_2015_83_MOESM1_ESM.docx]

**Additional file 1.** Bibliometric information associated with the other 80 articles of the T100.

| Rank | Author | Title | Journal | Year | Times Cited | PMID |  |
| --- | --- | --- | --- | --- | --- | --- | --- |
| 21 | Liu, J et al | Cathepsin L expression and regulation in human abdominal aortic aneurysm, atherosclerosis, and vascular cells | Atherosclerosis | 2006 | 104 | 15982660 |  |
| 22 | Gu, DF et al | Prevalence of cardiovascular disease risk factor clustering among the adult population of china - Results from the International Collaborative Study of Cardiovascular Disease in Asia (InterAsia) | Circulation | 2005 | 104 | 16043645 |  |
| 23 | Liu, Xifu et al | Neuregulin-1/erbB-activation improves cardiac function and survival in models of ischemic, dilated, and viral cardiomyopathy | Journal of the American College of Cardiology | 2006 | 104 | 17010808 |  |
| 24 | Tang, Yehua et al | MicroRNA-1 Regulates Cardiomyocyte Apoptosis by Targeting Bd-2 | International Heart Journal | 2009 | 102 | 19506341 |  |
| 25 | Chang, L et al | Protective effects of ghrelin on ischemia/reperfusion injury in the isolated rat heart | Journal of Cardiovascular Pharmacology | 2004 | 102 | 14716201 |  |
| 26 | Guo, XM et al | Creation of engineered cardiac tissue in vitro from mouse embryonic stem cells | Circulation | 2006 | 99 | 16651472 |  |
| 27 | Wei, Sheng et al | T-Tubule Remodeling During Transition From Hypertrophy to Heart Failure | Circulation Research | 2010 | 99 | 20576937 |  |
| 28 | Wu, Yangfeng et al | Estimation of 10-year risk of fatal and nonfatal ischemic cardiovascular diseases in Chinese adults | Circulation | 2006 | 97 | 17088464 |  |
| 29 | Hou, Mai et al | Transplantation of mesenchymal stem cells from human bone marrow improves damaged heart function in rats | International Journal of Cardiology | 2007 | 93 | 16889848 |  |
| 30 | Zhu, Ni et al | Endothelial enriched microRNAs regulate angiotensin II-induced endothelial inflammation and migration | Atherosclerosis | 2011 | 92 | 21310411 |  |
| 31 | Li, Shuqiang et al | Signature microRNA Expression Profile of Essential Hypertension and Its Novel Link to Human Cytomegalovirus Infection | Circulation | 2011 | 90 | 21690488 |  |
| 32 | Wang, YB et al | VKORC1 haplotypes are associated with arterial vascular diseases (stroke, coronary heart disease, and aortic dissection) | Circulation | 2006 | 89 | 16549638 |  |
| 33 | Lu, Zongliang et al | Effect of Xuezhikang, an extract from red yeast chinese rice, on coronary events in a Chinese population with previous myocardial infarction | American Journal of Cardiology | 2008 | 86 | 18549841 |  |
| 34 | Yang, Jinfu et al | Effects of myocardial transplantation of marrow mesenchymal stem cells transfected with vascular endothelial growth factor for the improvement of heart function and angiogenesis after myocardial infarction | Cardiology | 2007 | 85 | 16741354 |  |
| 35 | Yin, Yuehui et al | Prospective randomized study comparing amiodarone vs. amiodarone plus losartan vs. amiodarone plus perindopril for the prevention of atrial fibrillation recurrence in patients with lone paroxysmal atrial fibrillation | European Heart Journal | 2006 | 85 | 16825288 |  |
| 36 | Liu, ZG et al | Should the all elephant trunk" be skeletonized? Total arch replacement combined with stented elephant trunk implantation for Stanford type A aortic dissection | Journal of Thoracic and Cardiovascular Surgery | 2006 | 85 | 16399301 |  |
| 37 | Lin, Jiaxiong et al | Autonomic mechanism to explain complex fractionated atrial electrograms (CFAE) | Journal of Cardiovascular Electrophysiology | 2007 | 84 | 17916143 |  |
| 38 | Han, Yaling et al | Cilostazol in addition to aspirin and clopidogrel improves long-term outcomes after percutaneous coronary intervention in patients with acute coronary syndromes: A randomized, controlled study | American Heart Journal | 2009 | 83 | 19332203 |  |
| 39 | Sun, Chengbo et al | Advanced glycation end products depress function of endothelial progenitor cells via p38 and ERK 1/2 mitogen-activated protein kinase pathways | Basic Research in Cardiology | 2009 | 83 | 18622638 |  |
| 40 | Wang, J et al | Targeted disruption of Smad4 in cardiomyocytes results in cardiac hypertrophy and heart failure | Circulation Research | 2005 | 83 | 16151019 |  |
| 41 | Yang, Qi et al | Contrast-Enhanced Whole-Heart Coronary Magnetic Resonance Angiography at 3.0-T A Comparative Study With X-Ray Angiography in a Single Center | Journal of the American College of Cardiology | 2009 | 83 | 19555843 |  |
| 42 | Dong, Zhi Hui et al | Retrograde Type A Aortic Dissection After Endovascular Stent Graft Placement for Treatment of Type B Dissection | Circulation | 2009 | 82 | 19171859 |  |
| 43 | Chen, B. X et al | Neointimal coverage of bare-metal and sirolimus-eluting stents evaluated with optical coherence tomography | Heart | 2008 | 77 | 17923466 |  |
| 44 | Lu, Yanjie et al | MicroRNA-1 downregulation by propranolol in a rat model of myocardial infarction: a new mechanism for ischaemic cardioprotection | Cardiovascular Research | 2009 | 76 | 19581315 |  |
| 45 | Hu, Xinyang et al | Optimal temporal delivery of bone marrow mesenchymal stem cells in rats with myocardial infarction | European Journal of Cardio-Thoracic Surgery | 2007 | 76 | 17239611 |  |
| 46 | Zhou, Jing et al | Gradients of atrial refractoriness and inducibility of atrial fibrillation due to stimulation of ganglionated plexi | Journal of Cardiovascular Electrophysiology | 2007 | 76 | 17229305 |  |
| 47 | Chen, JZ et al | Effects of homocysteine on number and activity of endothelial progenitor cells from peripheral blood | journal of molecular and cellular cardiology | 2004 | 75 | 14871551 |  |
| 48 | Yang, Shilin et al | Cytochrome P-450 epoxygenases protect endothelial cells from apoptosis induced by tumor necrosis factor-alpha via MAPK and PI3K/Akt signaling pathways | American Journal of Physiology - Heart and Circulatory Physiology | 2007 | 71 | 17322420 |  |
| 49 | Sun, Ying-Gang et al | Hydrogen sulphide is an inhibitor of L-type calcium channels and mechanical contraction in rat cardiomyocytes | Cardiovascular Research | 2008 | 71 | 18524810 |  |
| 50 | Wang, Yibo et al | Polymorphisms of KDR gene are associated with coronary heart disease | Journal of the American College of Cardiology | 2007 | 71 | 17707181 |  |
| 51 | Liu, Jing et al | Ten-year risk of cardiovascular incidence related to diabetes, prediabetes, and the metabolic syndrome | American Heart Journal | 2007 | 70 | 17383293 |  |
| 52 | Liu, Tong et al | Statin use and development of atrial fibrillation: A systematic review and meta-analysis of randomized clinical trials and observational studies | International Journal of Cardiology | 2008 | 70 | 18031847 |  |
| 53 | Tang, Jun-Ming et al | VEGF/SDF-1 promotes cardiac stem cell mobilization and myocardial repair in the infarcted heart | Cardiovascular Research | 2011 | 68 | 21345805 |  |
| 54 | Yang, Yue-Jin et al | Atorvastatin treatment improves survival and effects of implanted mesenchymal stem cells in post-infarct swine hearts | European Heart Journal | 2008 | 68 | 18456710 |  |
| 55 | Zhang, Xue-Hua et al | New-onset heart failure after permanent right ventricular apical pacing in patients with acquired high-grade atrioventricular block and normal left ventricular function | Journal of Cardiovascular Electrophysiology | 2008 | 68 | 18005026 |  |
| 56 | Tang, Junming et al | Mesenchymal stem cells over-expressing SDF-1 promote angiogenesis and improve heart function in experimental myocardial infarction in rats | European Journal of Cardio-Thoracic Surgery | 2009 | 67 | 19524448 |  |
| 57 | Li, Kang et al | Differential Macrophage Polarization in Male and Female BALB/c Mice Infected With Coxsackievirus B3 Defines Susceptibility to Viral Myocarditis | Circulation Research | 2009 | 65 | 19608981 |  |
| 58 | Gao, Runlin et al | A Phase II, Randomized, Double-Blind, Multicenter, Based on Standard Therapy, Placebo-Controlled Study of the Efficacy and Safety of Recombinant Human Neuregulin-1 in Patients With Chronic Heart Failure | Journal of the American College of Cardiology | 2010 | 65 | 20430261 |  |
| 59 | Dong, Jia-Yi et al | Erectile Dysfunction and Risk of Cardiovascular Disease Meta-Analysis of Prospective Cohort Studies | Journal of the American College of Cardiology | 2011 | 65 | 21920268 |  |
| 60 | Kong, YZ et al | Macrophage migration inhibitory factor induces MMP-9 expression: implications for destabilization of human atherosclerotic plaques | Atherosclerosis | 2005 | 63 | 15585220 |  |
| 61 | Hou, Yinglong et al | Interactive atrial neural network: Determining the connections between ganglionated plexi | Heart Rhythm | 2007 | 63 | 17198991 |  |
| 62 | Shu, J et al | ST-segment elevation in the early repolarization syndrome, idiopathic ventricular fibrillation, and the Brugada syndrome: cellular and clinical linkage | Journal of Electrocardiology | 2005 | 63 | 16226071 |  |
| 63 | Chen, Shao-Liang et al | A Randomized Clinical Study Comparing Double Kissing Crush With Provisional Stenting for Treatment of Coronary Bifurcation Lesions Results From the DKCRUSH-II (Double Kissing Crush versus Provisional Stenting Technique for Treatment of Coronary Bifurcation Lesions) Trial | Journal of the American College of Cardiology | 2011 | 63 | 21329837 |  |
| 64 | Wang, Li et al | ADAMTS-7 Mediates Vascular Smooth Muscle Cell Migration and Neointima Formation in Balloon-Injured Rat Arteries | Circulation Research | 2009 | 62 | 19168437 |  |
| 65 | Cao, Feng et al | Long-term myocardial functional improvement after autologous bone marrow mononuclear cells transplantation in patients with ST-segment elevation myocardial infarction: 4 years follow-up double dagger | European Heart Journal | 2009 | 62 | 19508995 |  |
| 66 | Yan, Yuan et al | Bidirectional regulation of Ca2+ sparks by mitochondria-derived reactive oxygen species in cardiac myocytes | Cardiovascular Research | 2008 | 61 | 18006452 |  |
| 67 | Kang, Yu-Ming et al | Brain nuclear factor-kappa B activation contributes to neurohumoral excitation in angiotensin II-induced hypertension | Cardiovascular Research | 2009 | 61 | 19246475 |  |
| 68 | Zhou, Shuang et al | Repression of P66Shc Expression by SIRT1 Contributes to the Prevention of Hyperglycemia-Induced Endothelial Dysfunction | Circulation Research | 2011 | 61 | 21778425 |  |
| 69 | Zeng, Chunyu et al | Activation of D-3 dopamine receptor decreases angiotensin II type 1 receptor expression in rat renal proximal tubule cells | Circulation Research | 2006 | 61 | 16902178 |  |
| 70 | Lu, Zhibing et al | Atrial Fibrillation Begets Atrial Fibrillation Autonomic Mechanism for Atrial Electrical Remodeling Induced by Short-Term Rapid Atrial Pacing | Circulation- Arrhythmia and Electrophysiology | 2008 | 61 | 19808412 |  |
| 71 | Yang, Zhao-Ju et al | Prevalence of cardiovascular disease risk factor in the Chinese population: the 2007-2008 China National Diabetes and Metabolic Disorders Study | European Heart Journal | 2012 | 60 | 21719451 |  |
| 72 | Liao, Yu-Hua et al | Interleukin-17A Contributes to Myocardial Ischemia/ Reperfusion Injury by Regulating Cardiomyocyte Apoptosis and Neutrophil Infiltration | Journal of the American College of Cardiology | 2012 | 60 | 22261166 |  |
| 73 | Kang, S et al | Effects of statin therapy on the progression of carotid atherosclerosis: a systematic review and meta-analysis | Atherosclerosis | 2004 | 59 | 15530920 |  |
| 74 | Ma, Heng et al | Aldehyde dehydrogenase 2 (ALDH2) rescues myocardial ischaemia/reperfusion injury: role of autophagy paradox and toxic aldehyde | European Heart Journal | 2011 | 59 | 20705694 |  |
| 75 | Han, Yaling et al | Safety and Efficacy of Biodegradable Polymer-Coated Sirolimus-Eluting Stents in "Real-World" Practice 18-Month Clinical and 9-Month Angiographic Outcomes | JACC- Cardiovascular Interventions | 2009 | 59 | 19463441 |  |
| 76 | Liu, Xingpeng et al | Achievement of pulmonary vein isolation in patients undergoing circumferential pulmonary vein ablation: A randomized comparison between two different isolation approaches | Journal of Cardiovascular Electrophysiology | 2006 | 59 | 17239094 |  |
| 77 | Zhu, J. H et al | Homocysteine accelerates senescence and reduces proliferation of endothelial progenitor cells | Journal of molecular and cellular cardiology | 2006 | 58 | 16600290 |  |
| 78 | Cheng, TO et al | Real-time 3-dimensional echocardiography in assessing atrial and-ventricular septal defects: An echocardiographic-surgical correlative study | American Heart Journal | 2004 | 57 | 15632898 |  |
| 79 | Ma, H et al | Vasculoprotective effect of insulin in the ischemic/reperfused canine heart: Role of Akt-stimulated NO production | Cardiovascular Research | 2006 | 57 | 16212952 |  |
| 80 | Wu, XJ et al | Mesenchymal stem cells participating in ex vivo endothelium repair and its effect on vascular smooth muscle cells growth | International Journal of Cardiology | 2005 | 57 | 16274768 |  |
| 81 | Cao, Yu et al | Endothelial dysfunction in adiponectin deficiency and its mechanisms involved | Journal of molecular and cellular cardiology | 2009 | 57 | 19027750 |  |
| 82 | Zhou Wenwu et al | Limb Ischemic Preconditioning Reduces Heart and Lung Injury After an Open Heart Operation in Infants | Pediatric Cardiology | 2010 | 57 | 19787388 |  |
| 83 | Li, Hong-Liang et al | Targeted cardiac overexpression of A20 improves left ventricular performance and reduces compensatory hypertrophy after myocardial infarction | Circulation | 2007 | 56 | 17389268 |  |
| 84 | Luo, Wanjun et al | Effect of ischemic postconditioning in adult valve replacement | European Journal of Cardio-Thoracic Surgery | 2008 | 56 | 18078762 |  |
| 85 | Yin, F et al | Noncanonical cAMP pathway and p38 MAPK mediate beta(2)-adrenergic receptor-induced IL-6 production in neonatal mouse cardiac fibroblasts | Journal of molecular and cellular cardiology | 2006 | 56 | 16466739 |  |
| 86 | Kang, Yu-Ming et al | Cross-talk between cytokines and renin-angiotensin in hypothalamic paraventricular nucleus in heart failure: role of nuclear factor-kappa B | Cardiovascular Research | 2008 | 55 | 18469338 |  |
| 87 | Yu, Qiujun et al | Insulin says NO to cardiovascular disease | Cardiovascular Research | 2011 | 54 | 21051417 |  |
| 88 | Yang, Ruifang et al | Sodium tanshinone IIA sulfonate protects cardiomyocytes against oxidative stress-mediated apoptosis through inhibiting JNK activation | Journal of Cardiovascular Pharmacology | 2008 | 54 | 18427283 |  |
| 89 | Wang, B et al | Associations of plasma 8-isoprostane levels with the presence and extent of coronary stenosis in patients with coronary artery disease | Atherosclerosis | 2006 | 53 | 15996671 |  |
| 90 | He, Yao et al | Passive smoking and risk of peripheral arterial disease and ischemic stroke in Chinese women who never smoked | Circulation | 2008 | 53 | 18809795 |  |
| 91 | Zhang, Jing et al | Collagen-Targeting Vascular Endothelial Growth Factor Improves Cardiac Performance After Myocardial Infarction | Circulation | 2009 | 53 | 19307480 |  |
| 92 | Cui, Qi-Qiong et al | Assessment of atrial electromechanical coupling and influential factors in nonrheumatic paroxysmal atrial fibrillation | Clinical Cardiology | 2008 | 53 | 18257022 |  |
| 93 | Liu, J et al | Increased serum cathepsin S in patients with atherosclerosis and diabetes | Atherosclerosis | 2006 | 52 | 16140306 |  |
| 94 | Yan, Xiao Xiang et al | Increased serum HMGB1 level is associated with coronary artery disease in nondiabetic and type 2 diabetic patients | Atherosclerosis | 2009 | 52 | 19150066 |  |
| 95 | Zhang, Xiaohong et al | Spontaneous atherosclerosis in aged lipoprotein lipase-deficient mice with severe hypertriglyceridemia on a normal chow diet | Circulation Research | 2008 | 52 | 18032735 |  |
| 96 | Wu, Jiang-nan et al | Coffee consumption and risk of coronary heart diseases: A meta-analysis of 21 prospective cohort studies | International Journal of Cardiology | 2009 | 52 | 18707777 |  |
| 97 | Liang, DK et al | Finite element analysis of the implantation of a balloon-expandable stent in a stenosed artery | International Journal of Cardiology | 2005 | 52 | 16186062 |  |
| 98 | Chen, Shao-Liang et al | Comparison Between the NERS (New Risk Stratification) Score and the SYNTAX (Synergy Between Percutaneous Coronary Intervention With Taxus and Cardiac Surgery) Score in Outcome Prediction for Unprotected Left Main Stenting | JACC- Cardiovascular Interventions | 2010 | 52 | 20630456 |  |
| 99 | Zhao, Qiang et al | Randomized Study of Mononuclear Bone Marrow Cell Transplantation in Patients With Coronary Surgery | Annals of Thoracic Surgery | 2008 | 51 | 19021989 |  |
| 100 | Zhao, SP et al | Xuezhikang, an extract of cholestin, protects endothelial function through antiinflammatory and lipid-lowering mechanisms in patients with coronary heart disease | Circulation | 2004 | 50 | 15313947 |  |
